# Research on the Performance of Network Propagation by Using the Machine Learning and Internet-of-Things Technology Integrating Model

**DOI:** 10.1155/2022/5480015

**Published:** 2022-09-19

**Authors:** Feng Chen

**Affiliations:** College of Artificial Intelligence, Zhejiang College of Security Technology, Wenzhou 325016, Zhejiang, China

## Abstract

We combine machine learning with Internet of Things technology to study the performance of network propagation model. This paper first introduces the construction environment of the business push system and then realizes user clustering and active business push by using the experimental data. Experimental results show that the active service push system constructed in this paper is feasible and effective. The experiment also compares and analyzes the influence of different clustering methods on the accuracy of service push. The results show that the clustering effect of the multi-Markov chain model (m-MCM) method is superior to that of the *K-*means method, a commonly used machine learning method, and the accuracy of user-service push obtained by the m-MCM method is superior to that obtained by the *K*-means method. Finally, on the basis of the existing experimental results, the shortcomings of the service push system are summarized, the future improvement direction and specific implementation measures are proposed, and new requirements for the future update of the service push system are put forward.

## 1. Introduction

The industrial Internet of Things is the product of the deep integration of the Internet of Things, big data, cloud computing, and other new-generation information technologies into the manufacturing industry. It is the main starting point for the digital transformation of the manufacturing industry and has become a key support for the fourth industrial revolution. Germany, the United States, Japan, and other major developed countries have established promotion agencies for the industrial Internet of Things and formulated strategic plans for the development of advanced manufacturing industries, including the industrial Internet of Things, so as to realize the transformation and upgrading of traditional manufacturing industries and reshape their competitive advantages. China has also formulated the “Made in China 2025” policy to accelerate the in-depth integration of the new generation of information technology and manufacturing as the main line and to promote intelligent manufacturing as the main direction.

## 2. Related Works

The research on the industrial Internet of Things started earlier in the world, and the research from the perspective of economics mainly focuses on business model, innovation model, enterprise organization, and other fields. First, regarding research on the business model, Arnold et al. [1] divided the business model of the industrial Internet of Things into cloud-based business model, service-oriented business model, and process-oriented business model according to different service objects. Gierej [2] believes that the emergence of the industrial Internet of Things on the one hand heralds the prospect of the development of industrial intelligence and on the other hand marks the transformation of the traditional manufacturing industry to “Outcome Economy,” that is, the product and service delivery mode with customer demand as the core. Montori et al. [3] proposed that the industrial Internet of Things has spawned a new business model, which is due to the fundamental change of consumer behavior and the entry of competitors from outside the manufacturing industry (such as technology enterprises and information technology enterprises). In order to cope with the drastic change of market environment and the increasingly fierce competition, manufacturing enterprises must adjust their original product-centered business model to evolve into the business model of “integration of product and service” [4, 5]. Second, regarding research on the innovation model, Kazuyuki [6] analyzed the relationship between the utilization status, scope, and innovation of big data of Japanese B2B and B2C large enterprises based on questionnaire survey data and pointed out that the application of Internet of Things and big data can on the one hand improve enterprise productivity and on the other hand change enterprise production mode and work content, and the society needs to cope with and adapt to this change. Third, regarding enterprise organization research, Iivari et al. [7] pointed out that the key issues that industrial IoT enterprises need to consider are not only the technical level but also the organizational level so as to achieve the balance and adaptation of enterprise organization, human resources, and industrial IoT technology. This requires the corresponding adjustment of the organizational structure of the enterprise, such as the development and integration of industrial Internet technology, the reconstruction of the core business of the enterprise, and the new mode of production and employee incentive system. Since the beginning of the twenty-first century, academic research on platform enterprises has increased dramatically. The references research path is as follows [8, 9]: since 2000, bilateral market research [10, 11], platform architecture research [9], open innovation research [12], and business ecosystem research have transferred to user innovation research [8, 13], it has formed the research trend of platform strategy under the interdisciplinary background. As soon as the concept of cloud manufacturing was put forward, it became an emerging topic and was warmly welcomed by many scientific researchers. Based on cloud computing, Internet of Things, and other contemporary information technologies, cloud manufacturing extends the concept of “software as a service,” an emerging concept in the field of cloud computing, and is introduced into the manufacturing industry.

The research significance of information push technology is mainly reflected in three aspects. First of all, for service providers, a fast and accurate personalized service push system can improve the service quality, provide convenient and high-quality service content for service demanders smoothly, improve their competitiveness in the market, and promote the long-term development of enterprises. Secondly, for the service demand side, the intelligent service push technology saves users' time and energy and improves users' sense of pleasure in experience, and the efficient information push technology can realize the sharing value of information so that the majority of users can quickly find their service needs through this technology. Finally, for manufacturing production form, the development of information push technology accelerates the upgrade of the traditional manufacturing industry and makes manufacturing better integrated into the global economic development in order to improve the utilization rate of resources, reduce the manufacturing cost, promote the wisdom of the worldwide manufacturing model, and provide strong technical support [14].

From passive search to active screening and elimination, active service push technology has changed greatly the people's lifestyle. With the acceleration of social development, people hope to obtain more convenient and satisfying services through active service push technology. Information push is an active, intelligent, and perceptive information service form. According to the needs of users, the technology combines various advanced information technologies such as big data, artificial intelligence, and other technologies to timely and actively transfer information to the needs of users. This technology is widely used in e-commerce, social networks, information science, user personalized knowledge services, and other fields [15].

Active service push technology combined with automatic system recognition and analysis technology analyzes the potential interest preference of push objects. It helps manufacturing enterprises find potential service objects among a large number of users, realize real-time push operation of personalized service, and improve the accuracy and satisfaction of push service. Active push service is widely used in a web browser and e-commerce platform. For example, Baidu platform analyzes the frequency of browsing and length of stay according to the types of web pages frequently browsed by users and recommends news content that may be of interest to users according to the specific ranking order, thus eliminating the user input keywords or related website.

There are two characteristics of the active service push technology. (1) Active service push refers to the fact that the system automatically obtains the information of the web page browsing by users and uses various advanced data analysis technologies to push the content that users may be interested in. In the past, the demand analysis of services was initiated by users. (2) Cloud platforms are built through service virtualization. It is a gathering place of services and resources, realizing the sharing of services and resources, providing products and services needed by the market efficiently, and delivering them to users timely and accurately. Platform system 24 hours uninterrupted background operation.

Research institutions have done a lot of research on the combination of information push with business processes such as enterprise design and manufacturing. The existing research is mainly reflected in the application of knowledge push in specific fields [16]. In terms of active push of cloud services, Palmisano [17] explored the blank field of online e-retail system and designed and built a recommendation system based on environment awareness. Tosi [18] proposed a method of knowledge push based on user content after identifying the user's interest characteristics. Kobayashi et al. [19] proposed a search engine method based on user interest characteristics in the face of information flooding in the current network environment.

There is a lot of networked manufacturing service information in the cloud manufacturing service platform. This paper takes the data maintenance disaster caused by big data as the research background, analyzes the historical browsing data of users, and speculates users' preferences, tastes, and needs from the perspective of operators. A machine learning method based on the Markov chain model is proposed to aggregate users with the same interest characteristics, and then a collaborative filtering recommendation algorithm is used to analyze browsing logs, interaction logs, and transaction logs of users with the same interest characteristics. Finally, according to users' ratings, recommend some novel service information that users have not contacted before or may be interested in.

## 3. Construction of Network Propagation Model

This section introduces the peripheral architecture of the network propagation and how to use machine learning to realize the active push process of service.

### 3.1. The Peripheral Architecture of Network Propagation

**Figure 1:**
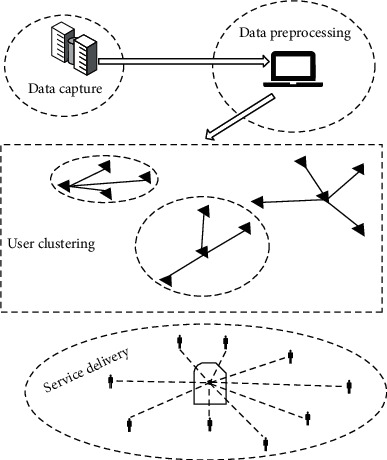
The peripheral framework of the push system.

As shown in Figure 1, the peripheral architecture of the push system is composed of four modules, which are data capture, data preprocessing, user clustering analysis with similar interest preferences, and service push module.

The main functions of each module in the push system are as follows.

#### 3.1.1. Data Capture Module

This module provides important user data resources for simulation experiments. The data acquisition of this module mainly includes two processes: (1) web crawler technology to simulate logging into a social manufacturing network to obtain interested topic database information; (2) web crawler technology combined with a website API interface to capture the information of many ordinary users in the platform. After a month of data collection, the web crawler helped obtain personal and browsing information from unauthorized users, eventually collecting 830 users and more than 200,000 text messages. In order to reduce potential errors, this paper divides the data set into 20% as test set and 80% as training set according to the rule of 2 : 8.

#### 3.1.2. Data Preprocessing Module

The module mainly refines and analyzes the obtained text information and converts the obtained text information into numerical information to enable computer recognition. This module mainly includes two processes. (1) Transform the interest topic library information into a standard user interest eigenvalue vector. (2) With the standard user interest eigenvalue vector as the reference, the text information of each user is transformed into the eigenvalue vector as the input parameter of machine learning.

#### 3.1.3. User Clustering Module with Similar Interest

This module mainly gathers a class of users with the same interest and hobbies together through the Markov chain model in machine learning, reduces the scope of user collaborative filtering calculation, and reduces the workload of the working platform. This module takes the user interest eigenvalue vector obtained in the data preprocessing module as the relevant parameters of the multi-Markov model. In the multi-Markov model, the eigenvalue vector of each user can be regarded as a Markov chain, so the set of users becomes the set of Markov chains. In the set of Markov chains, clustering and merging Markov chains with the same or similar characteristics can realize the aggregation of a class of users with the same interests.

#### 3.1.4. Service Push Module

This module is based on user clustering module with similar interests. Through the clustering module, a class of users with the same interest characteristics are gathered together. By analyzing the transaction record information obtained by the data acquisition module, the user-based collaborative filtering recommendation method is used to realize the active push of the service.

### 3.2. Overall Framework Design of Push System

**Figure 2:**
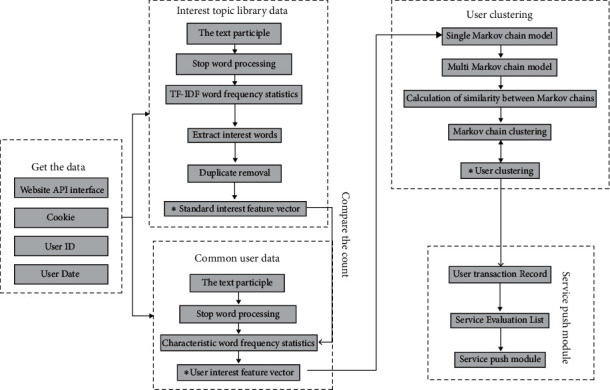
Push system internal details.

Section 3.1 outlines the peripheral architecture of the push system, and this section will analyze the main contents of the push system module one by one.  Figure 2 describes the main contents of the system in detail. Data acquisition module and data preprocessing module mainly include data acquisition, interest topic database data, and data processing of ordinary users. User clustering module mainly includes transforming user's eigenvalue vector into Markov chain and similar Markov chain clustering operation; service push module mainly includes service evaluation and active push operation of user transaction records. This section will introduce the main contents of user clustering module and service push module in detail.

### 3.3. Markov Chain Model

#### 3.3.1. Single Markov Chain Model (s-MCM)

According to the characteristics of the Markov chain, the interest characteristic value vector of each ordinary user is transformed into the corresponding parameters in the single Markov chain model to predict the user's interest orientation and interest category. However, if you want to use the single Markov chain model to judge the user interest category, you must make the following assumptions about the user browsing process on the website.

**Assumption 1 l.**

(Markov hypothesis) Assuming that the browsing process is a random process, users can be regarded as homogeneous discrete Markov chain. Therefore, a user's browsing record on the website is translated into a sequence of random variables *X*, which satisfies Markov property.

Under assumption 1, we establish a single Markov chain model (s-MCM).

**Definition 1 .**

s-MCM is represented by triple MC=〈*X*, *A*, *λ*〉 [20]. *X* represents the discrete random variable, its value range is {*x*_1_,…, *x*_*i*_,…, *x*_*n*_}, and *x*_*i*_ is one state of the model, representing that the user stays in the state of interest. *A* represents state transition probability matrix, which is also named transition matrix. In the transition matrix, the probability of transition from state *x*_*i*_ to state *x*_*j*_ can be calculated by the formula *P*_*ij*_=*P*(*X*_*t*_=*x*_*j*_/*X*_*t*−1_=*x*_*i*_), and the transition process can be simplified and written as a state pair (*x*_*i*_, *x*_*j*_). *λ* represents the initial state distribution, where each term *P*_*i*_=*P*(*X*_*t*=0_=*x*_*i*_). In summary, the transfer matrix *A* is

(1) $$\begin{array}{c} A=\left(p_{\mathrm{ij}}\right)=\left[\begin{array}{cccccc} P_{11} & P_{12} & \cdots & P_{1j} & \cdots & P_{1n}\\ P_{21} & P_{22} & \cdots & P_{2j} & \cdots & P_{2n}\\ \cdots & \cdots & \cdots & \cdots & \cdots & \cdots \\ P_{i1} & P_{i2} & \cdots & P_{\mathrm{ij}} & \cdots & P_{\mathrm{in}}\\ \cdots & \cdots & \cdots & \cdots & \cdots & \cdots \\ P_{n1} & P_{n2} & \cdots & P_{\mathrm{nj}} & \cdots & P_{\mathrm{nn}} \end{array}\right], \end{array}$$

(2) $$\begin{array}{c} R=\begin{array}{c} u_{1}\\ u_{2}\\ u_{3}\\ u_{4}\\ u_{5} \end{array}\left[\begin{array}{ccccc} 4 & 0 & 0 & 5 & 0\\ 5 & 5 & 0 & 5 & 4\\ 0 & 5 & 4 & 0 & 3\\ 5 & 3 & 5 & 5 & 4\\ 4 & 0 & 4 & 3 & 0 \end{array}\right]. \end{array}$$

At the same time, the initial state distribution is as follows:

(3) $$\begin{array}{c} \lambda =\left(p_{i}\right)=\left(P_{1},P_{2},\ldots ,P_{n}\right). \end{array}$$

According to the definition of the single Markov chain model, a user's interest eigenvalue sequence *X*_*i*_ is essentially a collection of all pages *x*_*i*_ visited by the user. By counting the number of visits that the user clicks on different pages *x*_*i*_, the vector of the user's number of visits to different pages is obtained, that is, the interest eigenvalue vector *X*_*i*_. All the data recorded in the process is denoted *d*_1_. By analogy, the data recorded by the *m* th user is *d*_*m*_, so the data set obtained by all users is *D*={*d*_1_, *d*_2_,…, *d*_*m*_}.

Thus, the parameters *P*_*ij*_ and *P*_*i*_ in the single Markov chain model based on all user data can be estimated by the data set *D* and the maximum likelihood estimation method; namely,

(4) $$\begin{array}{c} p_{\mathrm{ij}}=\frac{s_{\mathrm{ij}}}{\sum_{j=1}^{n}s_{\mathrm{ij}}}, \end{array}$$

(5) $$\begin{array}{c} p_{i}=\frac{\sum_{j=1}^{n}s_{ij}}{\sum_{i=1}^{n}\sum_{j=1}^{n}s_{ij}}. \end{array}$$

In (4) and (5), *s*_*ij*_ represents the number of occurrences of state pairs (*x*_*i*_, *x*_*j*_) in all user text information. When the parameters *p*_*ij*_ and *p*_*i*_ are calculated, substituted into (2) and (3), the triple parameters 〈*X*, *A*, *λ*〉 of the single Markov chain model can be obtained based on all user data.

#### 3.3.2. Multi-Markov Chain Model

As described in the summary of Section 3.3.1, it is inaccurate to describe all users' interest characteristics and categories only by establishing a single Markov chain model based on all users' data. In order to improve the accuracy of predicting user interest characteristics, this paper introduces the concept of multi-Markov chain model based on the single Markov chain model, which aims to obtain higher accuracy. The reason why the multi-Markov chain model is more suitable for the modeling of this topic is that the user's interest preference is affected by the user's own manufacturing environment, manufacturing capacity, and other factors. It is an uncertain complex sequence affected by multiple external factors. Therefore, the same category of users with the same or similar interest features is more suitable to be described by the same model; on the contrary, it is more reasonable to describe different types of users with different or not similar interest characteristics by different models. Based on this idea, a multi-Markov chain model based on all user data is established to describe the interest characteristics of all users. The premise is that the user's browsing process needs to meet the following two assumptions.

**Assumption 2 .**

(User Classification Hypothesis) Let all users have *K* categories, and then *C*={*c*_1_, *c*_2_,…, *c*_*k*_} denotes the category set of users, and *P*(*C*=*c*_*k*_) denotes the probability that any user belongs to category *c*_*k*_.

(6) $$\begin{array}{c} \sum_{k=1}^{K}P\left(C=c_{k}\right)=1. \end{array}$$

**Assumption 3 .**

(Markov-like chain hypothesis) Let the same category of users have the same or similar interest features, and the sequence of these features is a unique random process. The multi-Markov chain model is defined under Assumptions 1 and 2.

**Definition 2 .**

m-MCM is represented by four tuples 〈*X*, *K*, *P*(*C*), MC〉. *X* is a discrete random variable in the range of {*x*_1_,…, *x*_*i*_,…, *x*_*n*_}, and *x*_*i*_ is a state of the model, representing the user interest eigenvalues. *K* is a preset value in the model, representing the number of user classifications. *C*={*c*_1_, *c*_2_,…, *c*_*k*_} denotes the category of users, and the probability distribution of different types of users is represented by the distribution function *P*(*C*). Element *mc*_*k*_ represents the Markov chain composed of the same user *c*_*k*_ with the same interest type. If the element *mc*_*k*_ is set together, we obtain the class Markov set MC={*mc*_1_, *mc*_2_,…, *mc*_*k*_}, which represents the set of Markov chains of all categories of users.

In the multi-Markov chain model, the state transition matrix *A*_*k*_ of the class *k* is

(7) $$\begin{array}{c} A_{k}=\left(p_{kij}\right)=\left[\begin{array}{cccccc} P_{k11} & P_{k12} & \cdots & P_{k1j} & \cdots & P_{k1n}\\ P_{k21} & P_{k22} & \cdots & P_{k2j} & \cdots & P_{k2n}\\ \cdots & \cdots & \cdots & \cdots & \cdots & \cdots \\ P_{ki1} & P_{ki2} & \cdots & P_{kij} & \cdots & P_{kin}\\ \cdots & \cdots & \cdots & \cdots & \cdots & \cdots \\ P_{kn1} & P_{kn2} & \cdots & P_{knj} & \cdots & P_{knn} \end{array}\right]. \end{array}$$

And the initial distribution of class *k* is

(8) $$\begin{array}{c} \lambda_{k}=\left(p_{\mathrm{ki}}\right)=\left(P_{k1},P_{k2},\ldots ,P_{\mathrm{kn}}\right). \end{array}$$

In (7) and (8), the unknown parameters *p*_*kij*_ and *p*_*ki*_ can be obtained according to the definition of the multi-Markov chain. Because any user has its own unique interest characteristics in the initial state and can be regarded as a class of users alone, the parameters *p*_*kij*_ and *p*_*ki*_ can be obtained by a calculation method similar to the single Markov chain; namely,

(9) $$\begin{array}{c} P_{\text{kij}}=\frac{S_{\text{kij}}+a_{\text{kij}}}{\sum_{j=1}^{n}\left(S_{\text{kij}}+a_{\text{kij}}\right)}, \end{array}$$

(10) $$\begin{array}{c} p_{\mathrm{ki}}=\frac{\sum_{j=1}^{n}\left(S_{\mathrm{kij}}+\alpha_{\mathrm{kij}}\right)}{\sum_{i=1}^{n}\sum_{j=1}^{n}\left(S_{\mathrm{kij}}+\alpha_{\mathrm{kij}}\right)}. \end{array}$$

In (9) and (10), *S*_*kij*_ represents the number of state pairs (*x*_*i*_, *x*_*j*_) appearing in the class *k* user text information. *α*_*kij*_ is the superparameter. And the superparameter *α*_*kij*_ can be calculated by assuming that all state pairs (*x*_*i*_, *x*_*j*_) have the same number of occurrences, that is, Bayes assumption, and then *α*_*kij*_ can be obtained by (11) approximation:

(11) $$\begin{array}{c} \alpha_{\mathrm{kij}}=\frac{\beta }{n\times n}. \end{array}$$

In (11), constant *β* is often replaced by the size *n* of the problem domain space. In summary, when the parameters *p*_*kij*_ and *p*_*ki*_ are calculated, substituted into (7) and (8), we obtain the four-tuple parameters of the m-like Markov chain mode 〈*X*, *K*, *P*(*C*), MC〉. It is worth declaring that there are *m* users in the whole process, and the users are divided into *m* categories; each class Markov chain calculated represents a sequence of user interest eigenvalues.

### 3.4. Fusion of Interest Feature Vector and Multi-Markov Chain Model

The user's interest eigenvalue vector has been calculated in Section 3. This section will focus on how to use the known interest eigenvalue vector to calculate the parameters in the multi-Markov chain model.

The core idea is summarized as follows. Firstly, *K* = *m* is set to generate a quasi-Markov chain, and the practical significance of this step is to regard each user as an independent category, so the sequence of interest characteristic values of *m* users is correspondingly transformed into *m* Markov chains. Then, according to the one-to-one correspondence between *m* users and *m* Markov chains, the clustering operation of these Markov chains is carried out to realize the clustering of *m* users. The specific Markov-like chain generation process is as follows.

The initial state is regarded as a multi-Markov chain model under special circumstances; that is, a user's interest eigenvalue sequence is a category (*K* = *m*), and then the data set *D*={*d*_1_, *d*_2_,…, *d*_*m*_} composed of *m* user's interest eigenvalue is divided into K categories: *D*_1_, *D*_2_,…, *D*_*k*_. Therefore, each user category *c*_*k* is essentially a single Markov chain model based on learning data *D*_*k*. Therefore, the parameters *p*_*kij*_ and *p*_*ki*_ can be obtained by (9) and (10), and then the state transition matrix *A*_*k*_ and the initial state *λ*_*k*_ are obtained.

At this point, the interest eigenvalue vector of *m* users is transformed into a Markov-like chain of *m* users. Then, the clustering of similar or similar users is actually the clustering of similar or similar user class Markov chain.

### 3.5. Markov Chain Clustering

Section 3.4 has introduced in detail how the text data of *m* users are transformed into a Markov chain like *m* users; that is, the collected user data is transformed into a Markov chain set. The user clustering is actually the clustering of Markov chains, so the similarity evaluation criteria of two Markov chains must be defined in advance.

According to the definition, the dynamic characteristics of the Markov chain are represented by its state transition matrix, so the similarity between Markov chains *mc*_*k*_ and *mc*_l_ can be defined by mining the relationship between the two state transition matrices. The definition process of similarity is as follows. Let *mc*_*k*_ and *mc*_l_ be any two Markov chains, and their state transition matrices are *A*_*k*_ and *A*_l_, respectively. The *i* th row *p*_*ki*_ parameter in the state transition matrix *A*_l_ is *P*_*kij*_*|j*=1,2,…, *n*. And the *i* th row *p*_*li*_ parameter in the state transition matrix *A*_*k*_ is *P*_l*ij*_*|j*=1,2,…, *n*.

The parameters of both rows represent the distribution of variables [*t*] under a given condition *X*[*t* − 1]=*x*_*t*_, that is, *P*(*X*_*t*_*|X*_*t*−1_=*x*_*t*_), whose approximation can be represented by their cross-entropy. The parameters of the two lines *P*_*ki*_ and *P*_*li*_ both represent the distribution of the variable [*t*] under the given *X*[*t* − 1]=*x*_*t*_ condition, that is, *P*(*X*_*t*_*|X*_*t*−1_=*x*_*t*_), whose approximation can be represented by their cross-entropy:

(12) $$\begin{array}{c} \text{CE}\left(P_{\mathrm{ki}},P_{\mathrm{li}}\right)=\sum_{\text{j}=1}^{n}P_{kij}\log\frac{P_{kij}}{P_{lij}}. \end{array}$$

Therefore, the approximate degree of state transition matrices *A*_*k*_ and *A*_l_ can be expressed by the mean of cross-entropy of all row distributions; namely,

(13) $$\begin{array}{c} \text{Similarity}\left(A_{k},A_{\text{l}}\right)=\frac{\sum_{i=1}^{n}\text{CE}\left(P_{\mathrm{ki}},P_{\mathrm{li}}\right)}{n}. \end{array}$$

However, if (4)–(16) is directly used to evaluate the similarity between state transition matrices *A*_*k*_ and *A*_l_; there will be two defects:

1. First, the cross-entropy (12) is directional, so (13) does not meet the symmetric property; that is, Similarity(*A*_*k*_, *A*_l_) ≠ Similarity(*A*_l_, *A*_*k*_).
2. Equation (13) reflects that the more similar the dynamic characteristics of two Markov chains are, the smaller the similarity value of the two Markov chains is, which does not conform to the definition of similarity semantics.

Therefore, to avoid the above two defects, we define the similarity of *mc*_*k*_ and *mc*_l_ of two Markov chains as

(14) $$\begin{array}{c} \text{Similarity}\left(mc_{k},mc_{l}\right)=\frac{2}{\text{Similarity}\left(A_{k},A_{l}\right)+\text{Similarity}\left(A_{l},A_{k}\right)}. \end{array}$$

Note: if the Similarity(*A*_*k*_, *A*_l_)+Similarity(*A*_l_, *A*_*k*_)=0, 2/0 = ∞. The analysis (14) shows that the more similar the dynamic characteristics of two Markov chains are, the greater the similarity value is, which meets the requirement of similarity semantics.

### 3.6. Multi-Markov Chain Model Clustering Method

Based on the above steps, we give a user clustering method based on the multi-Markov chain model.

- User clustering steps:
- Input: user learning data *D*={*d*_1_, *d*_2_,…, *d*_*m*_}.
- Output: User clustering result.
- Process:
- Step l: Use (9) and (10) to map the learning data *D* into *M* Markov chains.
- Step 2: Use (11) to (14) to calculate the similarity degree between two Markov chains and arrange them according to the similarity value obtained by calculation from large to small.
- Step 3: According to the preset number of classifications and similarity values, cluster *m* Markov chains.
- Step 4: According to the corresponding relationship between *m* Markov chains and *m* users, output the clustering result.

### 3.7. Design of Active Service Push Algorithm

The service push system can be vividly described by a black box. The basic data resources of the pushed object are processed by the black box to obtain the push list, and they will receive service recommendations from the platform at a certain point offline.

In the existing service push system, the system that can achieve accurate push has not emerged because each push method only uses a small part of the system information. To achieve the goal of pushing more accurate, higher quality, and higher satisfaction services for service push objects, this paper adopts multilevel and all-round analysis methods to deal with the information acquired in the system, that is, the cluster-service collaborative filtering and mixed push method. Through the clustering analysis of users in the platform, users with the same interest category are classified into one class. Then, the collaborative filtering recommendation algorithm is used to push services to users with the same interest category. Because the evaluation model of “user-service“ for users of the same type is similar, there will not be too many nonevaluation services, which makes the evaluation matrix sparse. This can improve the accuracy of service push. The user clustering analysis method in the mixed push method has been introduced in detail in the previous sections, and the user-based collaborative filtering method will be explained in detail in the following.

### 3.8. User-Based Collaborative Filtering Recommendation Method

Through user clustering, a class of nearest neighbor user sets with similar interest characteristics can be obtained. The services in user sets are all closely related to users. Although the massive service types and quantity at the beginning of push have been reduced through clustering operation, service demanders will still be at a loss when facing a series of untargeted push services if these services are sent to service push objects in no order. In order to reduce the time spent by service demanders to understand each service, the nearest neighbor user set is substituted into the “user-service” evaluation model, which aims to generate a ranking list of services that users may like through this model. Finally, the service push object will receive the push information offline and in some way.

### 3.9. Establishment of “User-Service Evaluation Model”

“User-service evaluation model” is shown as follows. Assuming that there are *m* users in the nearest neighbor user set and *s* corresponding service sets, the “user-service” evaluation matrix *R* is

(15) $$\begin{array}{c} R=\begin{array}{cc} \begin{array}{c} u_{1}\\ \vdots \\ u_{i}\\ \vdots \\ u_{m} \end{array} & \begin{array}{c} \begin{array}{ccccc} r_{1} & \cdots & r_{j} & \cdots & r_{s} \end{array}\\ \left[\begin{array}{ccccc} R_{11} & \cdots & R_{1j} & \cdots & R_{s}\\ \vdots & \; & \; & \; & \vdots \\ R_{i1} & \cdots & R_{ij} & \cdots & R_{is}\\ \vdots & \; & \; & \; & \vdots \\ R_{m1} & \cdots & R_{mj} & \cdots & R_{ms} \end{array}\right] \end{array} \end{array}. \end{array}$$

In (15), UI represents the *i* th user, *Rj* represents the *j* th service, and *Rij* represents the score of the *i* th user on the *j* th service.

*Rij* is a real number ranging from 1 to 5. The higher the value of *Rij* is, the higher the user satisfaction is. If the user UI does not evaluate the service *Rj*, the value of *Rij* is defined as 0. The real-value scoring method is widely spread among service platforms because this scoring mode is more reasonable and detailed, which can greatly reduce the sparsity of “user-service” evaluation matrix *R* so as to improve the accuracy and satisfaction of service push to the greatest extent.

### 3.10. Similarity between Computing Services

When the “user-service” evaluation matrix *R* is used to calculate the list of push score values of similar services, cosine similarity value and Person similarity value between services can generally be calculated. However, the Person similarity calculation method is more delicate, and the average operation of subtracting the score value from the overall service score value is used to reduce the impact of different evaluation values on the same service with the same evaluation. The calculation results are more reliable and convincing, so the Person similarity measurement is adopted in this paper.

### 3.11. Person Similarity Measurement Calculation Method

Assuming that *I*_*pq*_ is the set of users who have commented on service *r*_*p*_ and *r*_*q*_ together in the nearest user set and $\bar{R_{p}}$ and $\bar{R_{q}}$ are the average ratings of users on service *r*_*p*_ and *r*_*q*_, respectively, then the similarity *δ*(*r*_*p*_, *r*_*q*_) is shown in (16):

(16) $$\begin{array}{c} \delta \left(r_{p},r_{q}\right)=\frac{\sum_{i\in I_{\mathrm{pq}}}\left(R_{\mathrm{ip}}-\bar{R_{p}}\right)\left(R_{\mathrm{iq}}-\bar{R_{q}}\right)}{\sqrt{\sum_{i\in I_{\mathrm{pq}}}{\left(R_{\mathrm{ip}}-\bar{R_{p}}\right)}^{2}}\sqrt{\sum_{i\in I_{\mathrm{pq}}}{\left(R_{iq}-\bar{R_{q}}\right)}^{2}}}. \end{array}$$

### 3.12. The Calculation Example of Service Actively Pushes

Calculation example 1: assume that users *u*_0_, *u*_1_, *u*_2_, *u*_3_, *u*_4_, *u*_5_ are a small group of nearest neighbor users after a clustering, and its user-service evaluation matrix is

(17) $$\begin{array}{c} \begin{array}{ccccc} r_{1} & r_{2} & r_{3} & r_{4} & r_{5} \end{array}\\ R=\begin{array}{c} u_{0}\\ u_{1}\\ u_{2}\\ u_{3}\\ u_{4}\\ u_{5} \end{array}\left[\begin{array}{ccccc} 4 & 0 & 0 & 5 & 0\\ 5 & 5 & 0 & 5 & 4\\ 0 & 5 & 4 & 0 & 3\\ 5 & 3 & 5 & 5 & 4\\ 4 & 0 & 4 & 3 & 0 \end{array}\right]\text{ .} \end{array}$$

Among them, $\bar{R_{1}}=3.67,\bar{R_{2}}=2.83,\bar{R_{3}}=2.67,\bar{R_{4}}=3.0,\bar{R_{5}}=1.83$. The users who jointly commented on service 1 and service 2 are *u*_1_, *u*_2_, *u*_4_, so *δ*(*r*_1_, *r*_2_) can be calculated by the following formula:

(18) $$\begin{array}{c} \delta \left(r_{1},r_{2}\right)=\frac{\sum_{i\in I_{12}}\left(R_{i1}-\bar{R_{1}}\right)\left(R_{i2}-\bar{R_{2}}\right)}{\sqrt{\sum_{i\in I_{12}}{\left(R_{i1}-{\bar{R}}_{1}\right)}^{2}}\sqrt{\sum_{i\in I_{12}}{\left(R_{i2}-{\bar{R}}_{2}\right)}^{2}}}\\ =\frac{\left(5-3.67\right)\times \left(5-2.83\right)+\left(4-3.67\right)\times \left(4-2.83\right)+\left(5-3.67\right)\times \left(3-2.83\right)}{\sqrt{{\left(5-3.67\right)}^{2}+{\left(4-3.67\right)}^{2}+{\left(5-3.67\right)}^{2}}\times \sqrt{{\left(5-2.83\right)}^{2}+{\left(4-2.83\right)}^{2}+{\left(3-2.83\right)}^{2}}}=0.7413. \end{array}$$

Furthermore, the calculation process of *δ*(*r*_1_, *r*_3_), *δ*(*r*_1_, *r*_4_),  and *δ*(*r*_1_, *r*_5_) is as follows:

(19) $$\begin{array}{c} \delta \left(r_{1},r_{3}\right)=\frac{\left(4-3.67\right)\left(4-2.67\right)+\left(5-3.67\right)\left(5-2.67\right)+\left(4-3.67\right)\left(4-2.67\right)}{\sqrt{{\left(4-3.67\right)}^{2}+{\left(5-3.67\right)}^{2}+{\left(4-3.67\right)}^{2}}\times \sqrt{{\left(4-2.67\right)}^{2}+{\left(5-2.67\right)}^{2}+{\left(4-2.67\right)}^{2}}}=0.9422,\\ \delta \left(r_{1},r_{4}\right)=\frac{\left(4-3.67\right)\times \left(5-3\right)+\left(5-3.67\right)\times \left(5-3\right)+\left(5-3.67\right)\times \left(5-3\right)+\left(4-3.67\right)\times \left(3-3\right)}{\sqrt{{\left(4-3.67\right)}^{2}+{\left(5-3.67\right)}^{2}+{\left(v\right)}^{2}+{\left(4-3.67\right)}^{2}}\times \sqrt{{\left(5-3\right)}^{2}+{\left(5-3\right)}^{2}+{\left(5-3\right)}^{2}+0}}=0.8908,\\ \delta \left(r_{1},r_{5}\right)=\frac{\left(5-3.67\right)\times \left(4-1.83\right)+\left(4-3.67\right)\times \left(3-1.83\right)+\left(5-3.67\right)\times \left(4-1.83\right)}{\sqrt{{\left(5-3.67\right)}^{2}+{\left(4-3.67\right)}^{2}+{\left(5-3.67\right)}^{2}}\times \sqrt{{\left(4-1.83\right)}^{2}+{\left(3-1.83\right)}^{2}+{\left(4-1.83\right)}^{2}}}=0.9819, \end{array}$$

where *δ*(*r*_1_, *r*_5_) > *δ*(*r*_1_, *r*_3_) > *δ*(*r*_1_, *r*_4_)*δ*(*r*_1_, *r*_2_) is sorted according to the service similarity value. And the active service push list is shown in Table 1.

Therefore, according to the service push data in Table 1, if user *u*_0_ is the active service push object, service 5 and service 3 will be pushed preferentially.

## 4. Experimental Analysis

### 4.1. Experimental Environment and Data

Experimental data set is a user data resource, which is obtained through the API interface provided by the official platform of China Machinery Network. In this paper, the web crawler software developed by myself crawls more than ten official users selected initially and extends other user data according to these users, realizing layer-by-layer crawler and deep mining. By the time of simulation analysis, about 830 users had been mined and recorded, who posted more than 200,000 SMS messages. In the face of such a large data set involving all aspects of society, it is crucial to correctly identify valid and invalid data. First, the distribution of the amount of text information published by these users is graphically described as follows.

**Figure 3:**
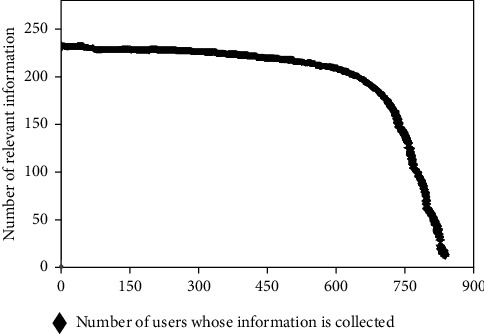
Distribution of the amount of published information of the NTH user.

As shown in Figure 3, the number of SMS messages sent by users is close to 248, and the number of similar users in this range is 478. When fewer than 200 messages were posted, the number of received messages dropped sharply, with the lowest users posting only about six messages. For example, the more detailed the user's information, the more accurate the classification of the user's interest characteristics, and vice versa. Therefore, in subsequent experimental tests, users with less information need to be filtered to improve the accuracy of user interest feature classification.

### 4.2. Experimental Evaluation Method

After the implementation of user clustering and service active push operation, this paper adopts the classic mean absolute error (MAE) as the measurement index to evaluate the accuracy of the recommendation system to verify its accuracy. MAE is an effective measure to evaluate the difference between the predicted score and the real score. Its basic idea is to calculate the difference between the predicted score and the real score and absolute value. After the absolute value operation, the evaluation indexes are all positive numbers, which avoids the offset between positive and negative values so as to reflect the deviation of predicted value error from the real score value to the greatest extent. The mathematical formula of MAE is defined as follows:

(20) $$\begin{array}{c} \text{MAE}=\frac{1}{N}\sum \left|P_{\mathrm{ij}}-R_{\mathrm{ij}}\right|. \end{array}$$

In (17), *N* represents the sum of the scores predicted by the test, *Pij* represents the predicted score value of user *i* for service *j*, *Rij* represents the real score of user *i* for service *j*. According to (17), the smaller the MAE value is, the smaller the predicted value error is and the higher the accuracy of recommendation is, and vice versa.

### 4.3. Experimental Analysis

#### 4.3.1. Data Dimension Reduction

**Figure 4:**
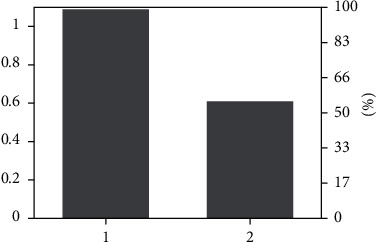
Pareto figure of user state transition matrix.

Section 3.2 introduces the processing method of interest subject database information and user text information in detail, and we obtain the interest eigenvalue vector of each user. In Section 4.3.2, the multi-Markov chain model is established to convert the eigenvalue vector of each user's interest into a unique corresponding state transition matrix, whose dimension is 624 × 624. Because each user has a unique Markov chain state transition matrix and the matrix dimension is 624 × 624, the clustering calculation will produce repeated data calculation and data dimension disaster, which will greatly affect the calculation efficiency. To sum up, in order to reduce the server load and improve the efficiency of calculation, this paper needs before the user clustering for all users of the Markov chain state transition matrix PCA (Principal Component Analysis) dimensionality reduction. The advantages of PCA dimension reduction are as follows: (1) data dimension reduction can alleviate dimensional disasters; (2) data dimension reduction can minimize information loss while compressing data; (3) the structure of data after dimensionality reduction is easier to understand through visualization. In short, the operation essence of PCA is to map the original data to the low-latitude numerical space through a series of numerical linear transformations under the premise that the characteristics and attributes of the original data are not damaged as much as possible.  Figure 4 is the Pareto figure of user state transition matrix.

#### 4.3.2. User Clustering

As described in Section 4.2, the accuracy of the user clustering effect in the early stage is directly related to the accuracy of user-service active push in the later stage. The clustering of users with the same or similar interest characteristics can increase the accuracy of the later service's active push. Therefore, this section mainly discusses the user clustering effects of the two methods. This paper adopts two methods to realize user clustering: (1) enhanced learning method in machine learning to realize user clustering, namely, multi-Markov chain model clustering method introduced in Section 3 (m-MCM clustering); (2) the unsupervised learning method in machine learning that realizes the clustering of users, namely, the classical *K*-means clustering method, the implementation of which is provided by Weka software. After user data processing, user clustering results are shown in Table 2.

**Figure 5:**
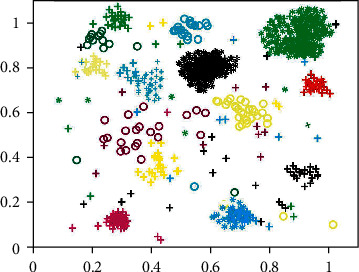
The effect of *K*-means user clustering method.

**Figure 6:**
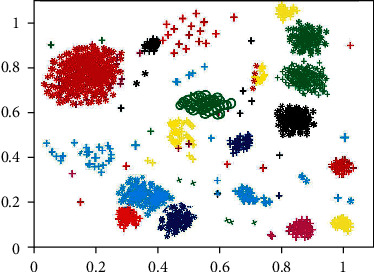
The effect of m-MCM means user clustering method.

As can be seen from Table 2, different clustering methods can obtain different clustering results; that is, there will be some deviation in the amount of clustering for each type of user. However, it is difficult to judge which clustering method has a better clustering effect only based on the data in Table 2. Therefore, Figures 5 and 6 are used to show the clustering situation of the *K*-means method and the m-MCM method for all users, respectively, so as to better distinguish the clustering effect of the two clustering methods. Note: each colored graph in the figure represents a user, a group of users with the same or similar characteristics of interest.

**Figure 7:**
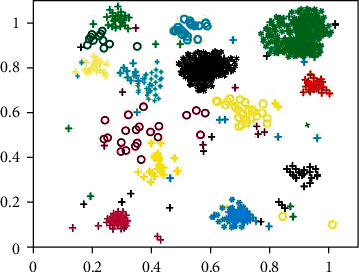
The effect picture of *K*-means user clustering method after denoising.

**Figure 8:**
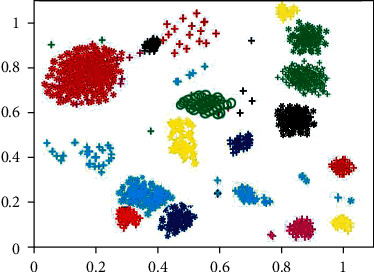
The Effect picture of m-MCM user clustering method after denoising.

By observing Figures 5 and 6, it can be found that the scattered noise points in the two images make the clustering effect picture very messy, and it is difficult to clearly distinguish the distribution of clustering users. These noise points are caused by the users with less published information mentioned in Section 3.10. Because the data of these users are less, it is difficult to accurately identify their interests, so they become noise points in the clustering effect figure. Because of these scattered noise points, it is difficult to distinguish the clustering of users accurately. At the same time, noise points will greatly reduce the accuracy of user clustering and affect the clustering effect. Therefore, noise point denoising is also adopted in this paper; that is, users with less data are deleted in advance. The user clustering effect figure after denoising is shown in Figures 7 and 8.

After denoising, Figures 7 and 8 show an obvious improvement in user clustering effect compared with Figures 5 and 6 without denoising. Although there are still noise points in Figures 7 and 8, the clustering of users can be distinguished, so the existence of some noise points is acceptable and within the error range. However, through observation, it can be found that there are significant differences in the user clustering effect in Figures 7 and 8. These differences come from the following: (1) In Figures 7 and 8, the clearance between cluster and the cluster is bigger, and it shows that other users have been spun off the accurate segmentation. On the contrary, in Figure 7, the gap between clusters is very small, and some clusters are even closely connected, which makes it difficult for us to determine which cluster these points close to the boundary belong to. Therefore, the m-MCM method has a better user clustering effect than the *K*-means method in the gap comparison between clusters and families. (2) As shown in Figure 8, within each family, the distance between points is mostly compact. On the contrary, in Figure 7, the distance between points inside each cluster is too scattered, which makes it difficult for us to distinguish some close clusters and determine whether this cluster is an aggregation of one category cluster or multiple category clusters. Therefore, the m-MCM method has a better user clustering effect than the K-means method in terms of gap comparison within clusters. (3) According to the clustering distribution in Figure 8, we can accurately divide the distribution of 20 types of user interest characteristics. On the contrary, according to the clustering effect in Figure 7, we can only roughly distinguish the user clustering distribution of 18 categories, which is a little deviation from the 20 categories of interest subject database that we set in advance.

**Figure 9:**
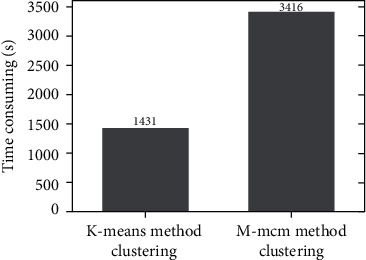
Comparison of clustering time between *K*-means method and m-MCM method.

In addition, Figure 9 lists the comparison figure of clustering time consuming of *K*-means method and m-MCM method. The figure shows that the clustering time of the m-MCM method is 3416 seconds, while that of the *K*-means method is 1431 seconds; that is, the clustering time required by the m-MCM method is about two times that of the *K*-means method. The m-MCM method is not dominant in the time consumed by clustering because the m-MCM method needs to repeatedly calculate the similarity between two Markov chains corresponding to 830 users in clustering. Therefore, although m-MCM clustering has a good clustering effect, it takes a huge amount of time.

To sum up, the m-MCM method is better than the *K*-means method in the user clustering effect. The m-MCM method can relatively accurately determine the interest category to which users belong and accurately define the boundary between classification and class, but it takes more time. If the m-MCM method is applied to the active push of user service, the accuracy of push can be greatly improved.

### 4.4. Verification of Collaborative Filtering Recommendation Method Based on User Score Clustering

**Figure 10:**
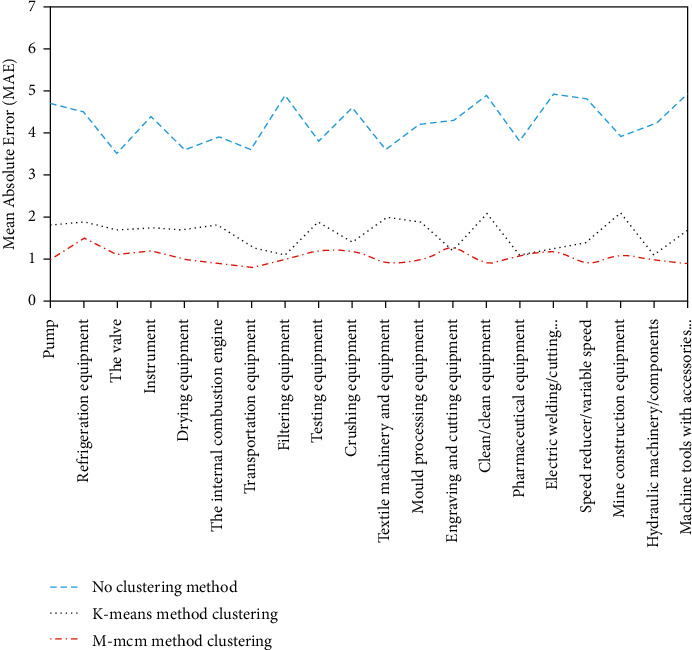
MAE under different clustering methods.

In order to verify the conjecture that the clustering operation in the early stage has a significant impact on the collaborative recommendation in the later stage, a series of simulation experiments are conducted to compare and evaluate the correctness of the conjecture based on the experimental results. Before the beginning of the experiment, the evaluation method of the experiment, data sources, and preprocessing methods should be explained in advance: (1) The evaluation method of the experiment is measured by MAE in Section 4.2. The smaller the MAE is, the higher the accuracy of the recommendation is. (2) The experimental evaluation results were measured according to the MAE value of each category. (3) The actual score in the experiment comes from the user's “user-service” evaluation matrix, while the predicted score is the service score calculated by the “user-service” evaluation model. (4) The scoring data of the experiment is about 160,000 service scoring data of 830 users in the social manufacturing platform. (5) The extended service set shall be considered after user clustering. If the original user does not have this service, the score of the extended service shall be set as 0. If the original user has a score for this service score, it will be added to the “user-service” evaluation matrix according to the original service score. As described in the previous chapter, the better and more accurate the user clustering effect in the early stage, the higher the accuracy of user-service push in the later stage. Therefore, three small experiments are designed in this section to verify this assumption: (1) without user clustering, collaborative filtering recommendation is carried out directly for users; (2) cluster users by *K*-means method, and then make collaborative filtering recommendation; (3) cluster users using the m-MCM method, and then make collaborative filtering recommendation. Experimental evaluation results are shown in Figure 10.

Through the analysis of Figure 10, the following conclusions can be drawn: (1) MAE value obtained by the nonclustering method is much larger than the MAE value obtained by the clustering method; that is, the accuracy of service push can be greatly improved by clustering users first and then actively pushing service to users. (2) The MAE value obtained by clustering with the m-MCM method is smaller than that obtained by clustering with the *K*-means method. In other words, different methods of clustering users will result in different accuracy of service push. At the same time, the better the clustering effect, the higher the accuracy of service push. (3) In comparison with MAE values of certain categories, the MAE values obtained by *K*-means clustering are smaller than those obtained by m-MCM clustering, which indicates that *K*-means clustering is also advantageous in some fields. If different clustering methods can be reasonably used in different fields to actively push users' services, in this way, a more precise and active service push effect can be achieved, which also provides a new research idea for future research. (4) As mentioned above, the user clustering takes a quantity, and the m-MCM method of clustering is not dominant. However, the time spent by M-MCM method is almost twice that of K-means method, but in return, the service push accuracy of M-MCM method is also close to twice that of K-means, and higher accuracy of service push will also consume more time. Therefore, these costs are acceptable in terms of time.

## 5. Summary and Discussion

The experiment in Section 4.2 implements user clustering and active service push. On this basis, the experiment also compares the clustering effect of the *K*-means method and the m-MCM method, as well as the accuracy of active service push. The experiment draws the following conclusions: (1) Cluster analysis of users before collaborative filtering can greatly improve the accuracy of active service push. (2) Different user clustering methods lead to different active service push. If the user clustering effect is better, the accuracy of the service push will be higher. Through experimental verification, this paper concludes that the clustering effect of the m-MCM method is better than that of the *K*-means method, and the accuracy of the clustering effect of the m-MCM method is better than the accuracy of service push after *K*-means method clustering. (3) In the early stage, spending more time on user clustering can improve the accuracy of the cluster, which is of great help to the later active service push. Since this paper constructed the active service push system for the first time, the experiment in Section 4.2 only preliminarily completed the basic architecture of active service push. In the future, the push system should be further updated and improved in the following aspects: (1) Reduce the clustering analysis time in the early stage of the push system and improve the clustering accuracy without reducing the user clustering effect. (2) A combination of multiple clustering methods is adopted to conduct cluster analysis on users so as to improve the speed and accuracy of cluster analysis and lay a good foundation for later service push. (3) The establishment of a more perfect “user-service” evaluation model can more accurately realize personalized service recommendation for users, thus improving the accuracy of service push by users.

## Figures and Tables

**Table 1:** Active service push list.

Push sequence sort	Service type	Service evaluation
1	*r*_5_ (service 5)	0.9819
2	*r*_3_ (service 3)	0.9422
3	*r*_4_ (service 4)	0.8908
4	*r*_2_ (service 2)	0.7413

**Table 2:** m-MCM and *K*-means clustering results.

Category	m-MCM	*K*-means
C_1	56	50
C_2	35	39
C_3	94	35
C_4	75	124
C_5	43	40
C_6	21	26
C_7	68	63
C_8	40	43
C_9	65	69
C_10	31	36
C_11	40	45
C_12	27	12
C_13	38	61
C_14	25	29
C_15	42	37
C_16	31	16
C_17	18	30
C_18	27	24
C_19	19	18
C_20	35	34

## Data Availability

The data can be obtained from the author upon request.
